# Subthreshold depression is associated with impaired resting-state functional connectivity of the cognitive control network

**DOI:** 10.1038/tp.2015.174

**Published:** 2015-11-17

**Authors:** J W Hwang, N Egorova, X Q Yang, W Y Zhang, J Chen, X Y Yang, L J Hu, S Sun, Y Tu, J Kong

**Affiliations:** 1 School of Acupuncture-Moxibustion and Tuina, Beijing University of Chinese Medicine, Beijing, China; 2 Department of Psychiatry, Massachusetts General Hospital, Harvard Medical School, Charlestown, MA, USA

## Abstract

Subthreshold depression (StD) is a prevalent condition associated with social morbidity and increased service utilization, as well as a high risk of developing into a major depressive disorder (MDD). The lack of well-defined diagnostic criteria for StD has limited research on this disorder, with very few brain-imaging studies examining the neurobiology of StD. Yet, identifying the neural pathology of StD has the potential to elucidate risk factors and prognostic markers for major depression and is crucial for developing tailored treatments for patients at mild stages of depression. We investigated resting-state functional connectivity (rs-FC) of the cognitive control network (CCN), known to be dysregulated in MDD, using the bilateral dorsolateral prefrontal cortex (DLPFC) as a seed, focusing on two cohorts of StD subjects (young and middle aged) as well as matched controls. Irrespective of age, we found a significant rs-FC decrease in the CCN of the StD subjects, compared with matched controls, particularly between the DLPFC and the brain regions associated with the representation of self and other mental states (temporo-parietal junction (TPJ) and precuneus), as well as salience detection and orienting (insula). The functional connectivity between the DLPFC and the left TPJ was also associated with depressive symptom scores measured by the Center for Epidemiologic Studies Depression Scale. This finding may shed light on the neural pathology of StD, leading to better understanding of mild stages of depression, its diagnosis and the development of new treatments.

## Introduction

Subthreshold depression (StD) refers to clinically relevant depressive symptoms that do not meet the criteria for a major depressive disorder (MDD).^1^ Although the symptoms of StD are less severe than the symptoms of MDD, StD is associated with a greater health service burden than MDD,^2^ due to its higher prevalence rate in population.^3, 4^ Moreover, individuals with StD are very likely to get a first-lifetime episode of MDD.^5^ Because of the lack of a clear definition and diagnostic criteria for StD, its etiology remains unclear.^6^ For the purposes of the current study, we followed the definition of StD suggested by Cuijpers *et al.*,^1^ which postulates that individuals with StD have clinically relevant depressive symptoms (assessed, for example, with a self-reported depression scale) but do not meet diagnostic criteria for a MDD, thus including individuals with mild depression but not patients with full-blown MDD. Intensive investigation of the neuropathology of StD is needed, as it will provide crucial information on the brain state during the medication-free stage of the depressive disorder, elucidating the dynamic course of depression-related brain changes from mild to major depression or recovery.

Cognitive impairment is one of the key characteristics of depressive patients and can have a severe impact on the depressed patient's ability to cope with the demands of daily living. It is reflected in both cognitive biases, that is, emphasis on negative emotions and self-focused thoughts, and cognitive processing problems, that is, lack of concentration, distractibility and memory problems.^7, 8, 9, 10^ The two are linked, as the habit of focusing on negative self-referential thoughts and feelings might cause impairment of attention, decision-making and conflict resolution.^11^ Cognitive deficits are a major reason for depressive individuals to seek treatment^5, 12, 13^ and therefore require careful investigation.

In the brain, depression-related cognitive impairment has been associated with structural and functional abnormalities in the regions pertaining to the cognitive control network (CCN).^14, 15^ The CCN includes fronto-parietal brain regions and is involved in top-down modulation of attention and working-memory tasks. Previous studies of MDD showed that depressive patients have dysregulated (some studies reporting increased^14^ but mostly decreased^16^) resting-state functional connectivity (rs-FC) in the CCN. Moreover, activation within the fronto-parietal network is associated with clinical outcomes and predicts treatment success. For instance, activation of the fronto-parietal network during the ‘no go' task significantly predicted remission following antidepressant treatment,^17^ and hypoconnectivity within the CCN predicted low remission rate and persistence of depressive symptoms,^18^ which further highlights the role of the CCN in depression and the importance of studying it.

Negative changes in cognitive functioning have been reported not only in MDD but also in StD subjects. Specifically, StD subjects were found to have limited integrative thinking and problem solving, as well as the use of inefficient cognitive strategies due to cognitive exhaustion, coupled with defocused attention.^19^ At the neural level, however, it remains unknown whether StD individuals exhibit similar pathophysiology of the CCN as the MDD patients.

In this study, we systemically investigated rs-FC in the CCN in two separate cohorts of first-episode StD subjects (young and middle aged) and compared them with healthy controls. We hypothesized that StD subjects would have impaired CCN rs-FC, especially in the brain regions associated with attention, memory and self-representation, similar to MDD patients. In addition, we examined any differences in CCN rs-FC between the young and middle-aged StD subject groups, as differences have been previously reported between younger and older adults' cognitive state potentially influencing their depression symptomatology.^20, 21, 22^

## Materials and methods

### Participants

In order to recruit StD subjects of different ages (young and middle-aged cohorts), we screened 981 subjects in universities or 383 subjects in local community centers (centers that provide programs, services and activities for local residents), respectively. All participants received a health lecture from investigators followed by a survey using the Center for Epidemiologic Studies depression scale (CES-D, Chinese version).^23^ The surveys were assessed by a trained clinician. Clinically depressed participants identified with the CES-D scale (score>16) and having a first episode of depression, were further assessed by a licensed psychiatrist using a 17-item Hamilton rating of depression scale (HAM-D) to confirm they qualify for the study in line with the inclusion/exclusion criteria below.

Inclusion criteria for StD participants were: (1) age 18–60 years; (2) CES-D score ⩾16, this cutoff score has been previously used to suggest the presence of clinical depression;^23, 24^ (3) 17-item HAM-D score of 7–17, corresponding to ‘mild' but not ‘no', ‘moderate' or ‘major' depression, in line with the typical descriptions of the severity levels of depression assessed with HAM-D.^25, 26, 27^ Exclusion criteria were (1) intelligence quotient ⩽90, measured with the Chinese version of Wechsler Adult Intelligence Scale^28^ based on the 1981 WAIS-R version; (2) diagnosis of severe depression by a licensed psychiatrist based on ICD-10; (3) prior use of psychiatric medications; (4) any suicidal tendencies posing immediate threat to the subject's life determined by a licensed psychiatrist in a clinical interview, based on the overall score of HAMD-17 questionnaire and specifically item 3 (suicide); and (5) major medical, neurological or psychological disorders, history of head trauma, or any functional magnetic resonance imaging (fMRI) contraindications, such as pregnancy or intent to become pregnant, claustrophobia and presence of metal in the body.

Healthy control participants were recruited from the same sources as StD participants based on the age and gender status of the selected StD participants and matched at a group level. Healthy controls did not have any history of depression and their CES-D scores were within the normal range (Table 1). All participants satisfied the inclusion criteria.

All participants were given a description of the study and were provided with the written informed consent forms. All subjects signed the consent forms before undergoing fMRI scans. The study was approved by the Committee on the Use of Human Subjects in Research at Beijing University of Chinese Medicine.

### MRI data acquisition

Images were acquired on a three-axis gradient head coil in a 3-Tesla Siemens (Beijing, China) MRI system equipped for echo planar imaging at the Research Institute of the State Key Laboratory of Cognitive Neuroscience and Learning at Beijing Normal University. Structural T1-weighted MRI sequence was followed by an 8-min resting-state scan. The T1-scanning parameters included repetition time of 2000 ms, echo time of 3.39 ms, flip angle of 70**°,** slices thickness of 1.33 mm; field of view of 256 mm^2^. For the resting-state fMRI, the scan acquisition included 32 slices with a thickness of 4.8 mm, repetition time of 2000 ms, echo time of 30 ms, flip angle of 90**°**, field of view of 240 mm^2^ and a 3 × 3-mm in-plane spatial resolution. During resting-state fMRI data acquisition, participants were instructed to remain still with their eyes closed and to let their minds wander freely.

### Seed-based functional connectivity analysis

The fMRI data were preprocessed using Data Processing Assistant for Resting-State fMRI (DPARSF) software (available at: http://rfmri.org/DPARSF)^29, 30^ implemented in a MATLAB suite (Mathworks, Natick, MA, USA). The software is based on Statistical Parametric Mapping (SPM8, http://www.fil.ion.ucl.ac.uk/spm) and Resting-State fMRI Data Analysis Toolkit (http://www.restfmri.net).^31^

The fMRI images were slice-timing corrected, head-motion corrected, coregistered to respective structural images for each subject and segmented; six rigid body motion parameters, white matter and cerebrospinal fluid signal were regressed out; the images were normalized by using structural image unified segmentation, and then resampled to 3-mm cubic voxels. After linear detrending, data were filtered using a typical temporal bandpass (0.01–0.08 Hz) to remove low-frequency noise (including slow scanner drifts) and the influence of higher frequencies reflecting cardiac and respiratory signals. Finally, the data were smoothed using a full width at half maximum of 6 mm.

Functional connectivity analysis for individual subjects was carried out in DPARSF by applying a seed-region approach. We used the seed applied in a previous study^14^ for elucidating the CCN network—the bilateral dorsolateral prefrontal cortex (DLPFC) (36, 27, 29, with 3 mm radius). This seed was chosen by Sheline *et al.*^14^ based on a previous study^15^ that identified the DLPFC as less active in depressed participants compared with controls during an emotion-interference conflict-matching task.

The averaged time course was obtained from the seed, and the correlation analysis was performed in a voxel-wise way to generate the FC map. The correlation coefficient map was converted into a Fisher-Z map by Fisher's r-to-z transform to improve the normality by calling functions in REST. To investigate the functional connectivity of CCN at a group level, individual Fisher-Z functional connectivity maps obtained from the functional connectivity analysis in DPARSF were used in the second-level analysis using SPM8 software. A full factorial design in SPM 8 was applied with factors age (young and middle age) and group (StD and control).

To explore the association between psychiatric measurements and rs-FC, we also performed regression analyses using the CCN rs-FC in all participants and the total CES-D score, including age and gender as non-interest covariates. A threshold of voxel-wise *P*<0.001 and cluster-level *P*<0.05 family-wise error corrected was used for all rs-FC analyses.

## Results

Of the 981 young subjects and 383 middle-aged subjects we screened, 57 subjects satisfied the criteria for StD. We recruited 79 healthy controls matching StD subjects for age and gender from the same population. All 136 subjects underwent the fMRI scan. The fMRI data from three healthy controls were excluded due to excessive head motion (greater than 3 mm during image acquisition), resulting in a total of 133 participants (76 healthy controls, 57 StD subjects) for the reported analyses.

The demographics of the two groups of participants are shown in Table 1. StD and healthy control groups did not differ significantly in terms of age, gender or years of education at a group level, except for the CES-D scores. CES-D scores did not differ between the young- and middle-aged StD groups.

### Resting-state functional connectivity results

**Figure 1:**
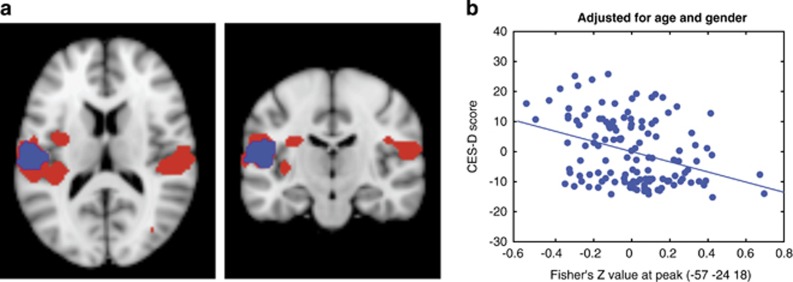
(**a**) Significant rs-FC results within the CCN. Red: significant differences between StD subjects and healthy controls (HCs>StD) in CCN connectivity; blue: significant association between the total CES-D score and rs-FC within the CCN. Overlap between the two analyses results in the left supramarginal gyrus/operculum. (**b**) Correlation between CES-D score and Fisher's *Z*-values at the peak of the significant cluster. CCN, cognitive control network; CES-D, Center for Epidemiologic Studies depression scale; HC, healthy control; rs-FC, resting-state functional connectivity; StD, subthreshold depression.

Functional connectivity analysis showed a significant CCN rs-FC decrease, specifically in the bilateral supramarginal gyrus (SMG)/operculum/superior temporal gyrus, left insula, precuneus and right parahippocampus in StD subjects compared with controls (Figure 1a). No significant differences between the young and middle-age groups were found in either StD subjects or healthy controls (Table 2), and no interactions between age (young vs middle) and group (depressed subjects vs controls) were observed.

Regression analysis between CES-D and CCN rs-FC in all subjects showed a negative association in the left SMG/operculum/superior temporal gyrus (peak coordinate: −57 −24 18, 190 voxels), overlapping with the group (StD<control) difference (Figure 1b). No positive association was observed.

## Discussion

In this study, we recruited two cohorts of StD subjects and matched controls to investigate whether CCN rs-FC in StD individuals of different ages differed from that of healthy controls. Bilateral DLPFC, previously shown to elucidate the CCN^14, 15, 18, 32^ was used as a seed. We found that rs-FC between the DLPFC and the bilateral SMG/operculum, left insula, precuneus and right parahippocampus was significantly decreased in StD individuals compared with controls. The rs-FC between the DLPFC and the left SMG/operculum was also negatively associated with the CES-D scores across all subjects. Our study demonstrates that rs-FC within the CNN is disrupted in StD subjects compared with healthy controls, and hypoconnectivity of DLPFC and left SMG correlates with severity of depressive symptoms in all subjects.

In agreement with the cognitive model of depression,^9, 33^ dysfunction of the cognitive processing (memory impairment, difficulty making decisions and loss of cognitive flexibility) and specific cognitive biases (concentrating on negative self-focused thoughts) are common among patients with MDD.^15, 34, 35^ Previous studies in MDD patients reported both increased^14, 36^ and decreased^18, 37^ rs-FC connectivity of the CCN in depressed patients compared with healthy controls, even when the same seed was used.^14, 18^ A recent meta-analysis of 25 rs-FC studies^16^ concluded that overall MDD is associated with hypoconnectivity within the cognitive control fronto-parietal network; decreased connectivity with the salience/emotion and attention networks; and hyperconnectivity with the default-mode network.

In line with these CCN rs-FC markers of MDD, we found that StD is also associated with an overall decrease within the CCN and its connectivity to the emotional and attention brain regions—in the SMG, precuneus, parahippocampal and insular regions. We also found an association between the CES-D scores and the connectivity of the DLPFC and left SMG in all subjects—the more depressed the subjects (including healthy controls) were, the lower connectivity was observed. Importantly, we compared young and middle-aged groups and found no differences in connectivity between the age groups. This result is consistent with previous studies reporting decreased fronto-parietal rs-FC in older depressed adults,^18^ middle-aged subjects^37^ and adolescent subjects who do not yet have depression but are at higher risk of MDD due to parental history.^32^ Together these findings suggest that changes in the rs-FC of the CCN are robust across age and severity of depression. We therefore speculate that decreased connectivity within the CCN, and especially between the DLPFC and left SMG (as we observed an overlap between the results of a group comparison and a regression with the CES-D scores), may be a potential marker of mild depression and a risk of developing MDD. Further longitudinal studies are needed to fully validate this claim.

The SMG is a brain region that could be relevant for both aspects of cognitive dysfunction in depression. On one hand, it is crucial for cognitive processing (attention and executive control). It has been suggested that the SMG is perfectly situated for convergence of the dorsal and ventral attention and/or memory streams, allowing attention reorientation to a salient stimulus.^38^ In a previous study,^39^ investigators found that prefrontal and parietal areas (including the SMG) were activated when executive control was required, suggesting that the connectivity of the two regions is crucial for executive control. On the other hand, SMG together with the angular gyrus belongs to the temporo-parietal junction (TPJ), which is relevant for cognitive biases (emotional (negative) and social (self-focus)). Previous studies suggested that the TPJ integrates information from both the external environment (obtained from visual, auditory and somatosensory systems) as well as from inside of the body.^40^ Researchers^40, 41^ also suggested that the right TPJ is involved in theory of mind processing, particularly in false-belief tasks, whereas the left TPJ (in combination with the frontal lobe) has an important role in the representation of the mental state.^42^ For example, a recent study^43^ combining behavioral tests, fMRI and transcranial magnetic stimulation, showed that the right SMG has an important role in overcoming emotional egocentricity bias in social judgments, which helps us distinguish our own emotional state from those of other people.

A decrease in functional connectivity of the prefrontal and supramarginal gyri has been previously reported in depression;^44^ it was negatively correlated with the ventral attention threat bias;^45^ and MDD patients exhibited a different pattern of brain network organization and specifically higher degree, that is, number of connections, in the left SMG.^46^ Given the functional importance of the SMG at the interface of cognitive and social processing and the results of our study identifying DLPFC/SMG connectivity significance in both analyses, the group comparison (StD vs control) and the regression with CES-D scores across all subjects, we speculate that the decreased rs-FC between the DLPFC and SMG may be primarily associated with the cognitive functions impairment (attention, memory and executive control), as well as cognitive biases experienced by the StD subjects such as increased self-focus and negative thoughts.^47^

Additional connectivity differences between StD and control groups involved the parahippocampus, insula and precuneus. Parahippocampus is a key region for memory function and is part of the limbic–paralimbic system. Previous studies found decreased hippocampal connectivity to frontal regions, which correlated with depression symptoms, specifically in StD subjects.^48^

Left anterior insula is a part of the CCN,^49^ which specifically belongs to the salience network involved in detecting and orienting to both external and internal salient stimuli and events.^50^ It is also part of the affective network and as such is important for both cognitive functions and cognitive biases. Using the surface-based regional homogeneity method,^51^ Li *et al.*^52^ found that compared with healthy controls, first-episode drug-naive MDD patients showed reduced surface-based regional homogeneity in the left insula. MDD has also been characterized by the decreased connectivity between the insula and subgenual ACC,^53^ as well as decreased bilateral amygdala and the left insula within the affective network.^37^ Thus, decreased rs-FC between the DLPFC and the insula may represent impaired cognitive control and salience detection in StD subjects, as well as impaired emotional processing.

Finally, precuneus is a key node of the default-mode network,^54, 55, 56^ as well as part of the theory of mind network^57^ involved in emphatic judgements.^58^ Accumulating evidence^59, 60, 61, 62, 63, 64, 65^ suggests that the default mode network has an important role in neuropathology of depression, and may underlie the abnormal self-referential processing in depressed patients. On the basis of a model presented by Abu-Akel and Shamay-Tsoory^40^ self and other mental states are formed in the TPJ, and are then relayed through the superior temporal gyrus or the precuneus/posterior cingulate complex to the limbic–paralimbic regions to be assigned cognitive or affective values. Thus, we believe that impaired functional connectivity between the DLPFC and the key regions of the mental-state network (including the left TPJ/SMG and left precuneus) may underlie cognitive processing problems and maladaptive cognitive biases in StD individuals.

It is worth noting that there is much variability in the definitions of StD^6^ across studies. Although some investigators only include subjects in preclinical stages of depression (for example, CES-D score 8–15,^66^ which is below the clinical level of 16), many others^1, 67^ by definition require the presence of clinically relevant symptoms and include subjects with clinical mild depression preceding MDD, as we did in the current study. This variability of the definition of StD might result in heterogeneity of the imaging findings. Therefore, one should pay close attention to the definition of ‘subthreshold' depression and specific inclusion criteria used for subject recruitment, when comparing results of different studies.

### Limitations

We here focused on the differences between healthy and StD population in the rs-FC of the CCN. Although the rs-FC pattern seems similar between MDD and StD, based on existing MDD findings and our StD results, future studies should directly explore differences in CCN between StD and MDD individuals. With some evidence pointing to StD as a risk factor for developing MDD,^1^ it could be useful to investigate neural signatures of transition between mild and moderate and severe depression, albeit in a longitudinal study following StD subjects to recovery or full-blown MDD. The regression analysis results across healthy and StD subjects in the current study, however, suggest that the connectivity of the DLPFC and the left SMG could potentially signal transition from mild to major depression. In the current study, we only controlled for such variables as age, gender, history of depression and years of education; however, other factors possibly contributing to the disturbances in the CCN, such as anxiety level, require further investigation.

In summary, we found a significant rs-FC decrease within the CCN, and specifically between the DLPFC and brain regions associated with self and other mental states (SMG/TPJ, precuneus), attention and executive control (SMG), salience detecting and orienting (insula) and memory (hippocampus) in StD subjects, compared with matched controls. This neural signature of StD is similar to MDD, and signals problems with both cognitive processing and cognitive biases typically observed in depression. This finding may shed light on the brain pathophysiology of depression in a mild stage of the disorder.

## Figures and Tables

**Table 1:** The demographic variables of the subjects with StD and demographically matched (at the group level) healthy control subjects

	*Items*	*Healthy control group*	*StD group*	P-*value*
All subjects	*N* (male)	76 (23)	57 (15)	0.700
	Age (years) (mean±s.d.)	29.86±14.49	32.25±15.62	0.364
	Education (years) (mean±s.d.)	14.77±3.80	15.35±3.00	0.321
	CES-D (mean±s.d.)	5.96±4.16	25.72±6.02	0.000
	HAM-D (17 items) (mean±s.d.)	NA	10.65±2.69	—
Young age	*N* (male)	51 (18)	34 (12)	0.590
	Age (years) (mean±s.d.)	20.63±1.89	20.29±1.40	0.333
	Education (years) (mean±s.d.)	15.96±2.92	16.32±2.40	0.533
	CES-D (mean±s.d.)	6.78±4.17	24.56±6.66	0.000
	HAM-D (17 items)	NA	9.56±2.02	—
Middle age	*N* (male)	25 (5)	23 (3)	0.400
	Age (years) (mean±s.d.)	49.20±10.25	49.91±8.44	0.795
	Education (years) (mean±s.d.)	12.32±4.27	13.91±3.27	0.156
	CES-D (mean±s.d.)	4.40±3.58	27.43±4.55	0.000
	HAM-D (17 items) (mean±s.d.)	NA	12.04±2.84	—

Abbreviations: CES-D, Center for Epidemiologic Studies Depression Scale; HAM-D, Hamilton rating of depression scale; NA, not applicable; StD, subjects with subthreshold depression.

**Table 2:** Brain regions showed significant CCN rs-FC differences between all StD subjects (young and middle age) and healthy controls

	*Region*	*Coordinates*	*Peak Z*	*Cluster size*
		*(x,y,z)*		
HC>StD	R parahippocampus	15,−36,−15	4.61	176
	R fusiform gyrus	21,−48,−15	4.30	
	L supramarginal gyrus/operculum	−57,−24,15	4.41	637
	L insula	−36,−27,12	3.73	
	R supramarginal gyrus/operculum	54,−24,15	4.40	257
	Left precuneus	−12,−54,66	4.05	231
StD>HC	None			

Abbreviations: CCN, cognitive control network; HC, healthy control; L, left; R, right; rs-FC, resting-state functional connectivity; StD, subthreshold depression.
